# The relationship between postsecondary education and adult health behaviors

**DOI:** 10.1016/j.ssmph.2021.100992

**Published:** 2021-12-03

**Authors:** Anthony Jehn

**Affiliations:** University of Western Ontario, Social Science Centre, Room 5225C, 1151 Richmond Street, London, Ontario, N6G 2V4, Canada

**Keywords:** Health behaviors, Postsecondary education, Adults, Gender, Race, United States

## Abstract

Nearly 80% of American adults between the ages of 33-44 have at least some postsecondary education, which ranges from vocational training to a doctorate or professional degree. However, in education-health studies, postsecondary credentials are often grouped into a limited number of categories. This is an important omission as it obscures differentiations between the various types of postsecondary credentials. This study provides the first comprehensive analysis of disparities in health behaviors across detailed levels of postsecondary education. Data comes from Wave 5 of the 2018 National Longitudinal Study of Adolescent to Adult Health (Add Health). A covariance-weighting technique is used to produce behavioral index scores that identify the full spectrum of health behaviors influenced by postsecondary educational attainment. Estimates are initially produced in aggregate for the total sample population, with interaction models subsequently being used to test differences across gender and race/ethnicity population subgroups. The aggregate results indicate that adults with at least a bachelor's degree exhibit healthier lifestyles; however, no difference is observed among adults with lower-level postsecondary credentials, compared to high school graduates. Women experience steeper gradients at higher levels of postsecondary education, compared to men. Both White and Hispanic American adults exhibit comparable health lifestyles across levels of postsecondary education; however, Black Americans were found to experience no returns except at the doctorate or professional degree level. These findings have important implications particularly as adults in their thirties and forties continue to exhibit troubling health and mortality trends.

## Highlights

• Adult health behaviors across detailed levels of postsecondary education.
• Estimates are provided both in aggregate and by the most influential population subgroups, including gender and race.
• Significant better health behaviors found among BA graduates and above.
• Lower or no returns found among sub-BA holders.

## 1 Introduction

A postsecondary education is a fundamental social determinant of adult health behaviors (Johnson et al., 2016, p. 369; Link and Phelan 1995; Schüz et al., 2020). However, education-health studies often group postsecondary credentials into a limited number of categories such as “some postsecondary” and “completed college or university” (Case & Deaton, 2021; Jehn and Zajacova 2019; Lawrence, 2017; Zheng, 2017). This is an important omission as it obscures differentiations between the various types of postsecondary credentials, particularly as nearly 80% of adults between the ages of 33-44 have at least some postsecondary education. The expansion of higher education has also led to a diversified number of available postsecondary credentials, which range from vocational training to a doctorate or professional degree. These diversified credentials represent profoundly different levels of human capital accumulation, which lead to comparably varied life trajectories. Therefore, aggregating millions of adults with various levels of educational attainment into a limited number of categories obscures the potential magnitude of disparities across the adult population. The present study thus examines associations between a postsecondary education and adult behaviors using detailed levels of educational attainment.

To better understand associations between postsecondary educational attainment and adult health behaviors, this study adopts the health lifestyles theoretical framework. Developed by Cockerham et al. (1997), health lifestyles are defined as a combined pattern of health behaviors based on available options according to social conditions and individual life chances. Individuals with similar status and class distinctions form aggregate status groups and share similar lifestyles (Burdette et al., 2017). For example, status groups influence our exposure to various health related norms and customs through socialization and shared experiences. Level of education, gender, and race/ethnicity are important factors for the formation of status groups, each of which are associated with unique sets of health behaviors that have variable impacts on overall health and well-being (Mollborn et al., 2014; Ross et al., 2016). To assess adult health lifestyles, this study combines some of the leading behavioral causes of early mortality to identify the full spectrum of behaviors associated with postsecondary educational attainment.

Due to the differential returns to a postsecondary education, I also examine disparities across the most influential demographic characteristics including gender and race/ethnicity. The theories of resource substitution and multiplication present two competing hypotheses for assessing disparities in health-related education returns across population subgroups (Ross & Mirowsky, 2006). Resource substitution suggests individuals who are marginalized within society will experience steeper education-health gradients as they have fewer alternative health-promoting socioeconomic resources (Ross & Mirowsky, 2010; Vable et al., 2018). For example, through a process of resource substitution, changing patterns of depression were found to depend more strongly on additional years of education for women than for men (Ross & Mirowsky, 2006). In contrast, resource multiplication theory suggests education-health returns are greater among socially advantaged population subgroups (Ross & Mirowsky, 2006). Advantaged groups gain more from education as their social and economic resources multiply to perpetuate and enhance their advantage (Montez & Barnes, 2016). As such, examining the potential differences by gender and race/ethnicity may help us to better understand the relationship between postsecondary education and adult health behaviors.

### 1.1 Differences in education returns

The knowledge and skills acquired through a postsecondary education can impact health behaviors indirectly through better occupations and incomes as well as directly by enhancing cognitive and non-cognitive abilities to enable healthier lifestyles (Carroll et al., 2017). Adults with higher levels of education generally demonstrate healthier behaviors including lower rates of smoking and alcohol consumption, increased physical activity, and better nutrition (Collin et al., 2021; Ettman et al., 2020; Jamal et al., 2018). Higher education is also associated with a greater use of safety and preventative health measures (Cutler & Lleras-Muney, 2010; Pampel et al., 2010). The positive education-health gradient is largely thought to be universal across populations, health outcomes, and across the entire range of educational attainment (Conti et al., 2010; Mirowsky & Ross, 2017).

Although educational attainment remains one of the strongest social determinants of adult health behaviors, a growing body of literature has shown that additional levels of education do not uniformly equal better health. For example, several recent studies have found that adults with some college but no degree and those with technical/vocational associate degrees report more pain and a higher prevalence of a broad range of health conditions than high school graduates who never attended college (see Zajacova & Johnson-Lawrence, 2016; Zajacova & Lawrence, 2021; Zajacova et al., 2012). Adults with a bachelor's degree were also found to have better physical functioning at mid-life, compared to high school graduates, while no returns were observed among those with lower-level postsecondary credentials (Carroll et al., 2017). These critical differences in postsecondary outcomes motivate the need to examine adult health behaviors across the entire education gradient.

### 1.2 Gender and Race

Gender influences labor market outcomes and economic returns to a postsecondary education. As a result, men and women may also exhibit significant differences in their health behaviors. For example, regardless of their level of education, men generally exhibit higher instances of substance abuse and other adverse health-related behaviors than women (Hill et al., 2006; Olson et al., 2017; Ross et al., 2016). Conversely, women more effectively adopt positive health behaviors, including proper nutrition and preventative care. There is also some evidence to suggest women experience steeper education-health gradients, which supports a process of resource substitution; however, resource multiplication theory would predict that men gain more from education than women due to their social and economic advantages (Ross & Mirowsky, 2010; Vable et al., 2018). Therefore, the complexity of gender differences in health behaviors deserves attention, particularly as adults in their thirties and forties continue to exhibit troubling health and mortality trends and whose most productive years of work and family life are ahead of them.

The association between a postsecondary education and adult health behaviors may also differ by race/ethnicity. Black Americans earning at least a bachelor's degree continue to be exposed to higher levels of stress and disadvantages in a society where they suffer social, economic, and political exclusion (Simons et al., 2020). For example, among college graduates, the Black American unemployment rate is approximately two-thirds higher and their salaries are substantially lower than their non-Hispanic White counterparts (Brown, 2019). Hispanic/Latino adults also experience economic disadvantages, compared to non-Hispanic White Americans (Goldman et al., 2006; Turra & Goldman, 2007; Vable et al., 2018). Lower economic returns to a postsecondary education among Black and Hispanic/Latino Americans causes socioeconomic disadvantages such as residing in impoverished neighborhoods and poor access to health care (Cutler & Lleras-Muney, 2006; Warren et al., 2020). As a result, it is reasonable to expect that lower income, incidents of personal discrimination, and living in disadvantaged neighborhoods determine exposure to health-related norms and customs which negatively influence adult health behaviors. On the other hand, one would expect to finder steeper education-health gradients among Black and Hispanic/Latino Americans as they have fewer alternative health-promoting socioeconomic resources. These conflicting possible outcomes motivate the need to examine racial differences in adult health behaviors.

### 1.3 Contributions of this study

The potential differences in the association between a postsecondary education and adult health behaviors necessitate the need for a comprehensive analysis of disparities across the entire postsecondary education gradient. My aim is to contribute to this literature by not only providing the first systematic analysis but to also identify important differences both in aggregate and across gender and race/ethnicity population subgroups. Using a covariance-weighting technique, I assess adult health lifestyles using a weighted behavioral index that combines some of the leading behavioral causes of early mortality to identify the full spectrum of behaviors associated with postsecondary educational attainment. My analysis reveals substantial disparities in adult health behaviors even after taking into account a range of potential confounders and mechanisms relevant to the education-health behavior association. This includes various additional demographic characteristics as well as measures of socioeconomic status.

## 2 Methods

### 2.1 Data

Data comes from Wave 5 of the 2018 National Longitudinal Study of Adolescent to Adult Health (Add Health), administered by the University of North Carolina Population Center. The purpose of the survey was to collect information about respondents’ social behaviors, economic resources, as well as their physical and mental wellbeing. Wave 5 of Add Health includes a nationally representative sample of mid-life adults between the ages of 33-44 in the United Sates. Data were collected between 2016 and 2018. The sampling frame includes all Add Health respondents who have been followed from adolescence (grades 7–12) into adulthood with a total sample size of 12,300 respondents.

Due to the large number of health behavior-related questions, Add Health data is uniquely positioned to offer insights about the associations between a postsecondary education and adult health behaviors. The data also covers the ideal age range, as it reflects the period immediately following the transition to adulthood where health behaviors are better established (Lawrence, 2017). The analytic sample is defined as adults 33 to 44 years of age. Respondents were excluded if they did not have valid responses about their educational attainment or for any of the health behaviors assessed in this study. The final analytic sample includes 11,560 respondents.

### 2.2 Measures

#### 2.2.1 Outcome variables

This study examines a total of six adult health behaviors including binge drinking, smoking, marijuana use, physical activity, fast food consumption, and obesity. Binge drinking measures the number of alcoholic beverages respondents usually have each time they had drinks in the last 30 days. Those having more than four (women) or five (men) are considered binge drinkers. Smoking and marijuana use are operationalized as dichotomous measures identifying respondents who have smoked tobacco or marijuana, respectively, in the past 30 days. Physical activity assesses how many times per week a respondent participates in six different categories of activities for exercise, coded with a range of 0 to 42. Fast food consumption measures how many times respondents ate fast food in the last 7 days, with a range of 0 to 50. Both variables are dichotomized at their midpoint to identify respondents with below average levels of activity or respondents that consume an above average level of fast food, respectively. Obesity is a dichotomous measure of the proportion of respondents experiencing a body mass index ≥30. While obesity is not a direct health behavior, it is an important biomarker proxy of nutritional habits (see Knol et al., 2017; Lawrence, 2017; Price et al., 2017; Rigdon et al., 2017; Shinde, 2019). Once all outcome variables are properly specified, I constructed a health behavior summary index using a covariance-weighted average of indicators as proposed by Anderson (2008). The primary advantage of this procedure is that it increases efficiency by ensuring highly correlated indicators receive less weight than uncorrelated indicators (Schwab et al., 2020). Higher index scores indicate increased engagement in unhealthy behaviors. A further description of these variables is provided in Table 1.

**Table 1:** Measures used to generate weighted health behavior index scores.

Variables	Description
Binge Drinking	Continuous measure identifying the number of alcoholic beverages respondents usually have each time they had drinks in the last 30 days (range: 0–99).
Smoking	Continuous measure identifying the number of cigarettes respondents usually smoke each day in the last 30 days (range: 0–300).
Marijuana Use	Ordinal measure indicating the number of days respondents used marijuana in last 30 days: (0) never; (1) one day; (2) 2 or 3 days; (3) 1 day a week; (4) 2 days a week; (5) 3 to 5 days a week; (6) every day or almost every day
Physical Activity	Continuous measure of bouts of physical activity across six broad categories in the last 7 days (range: 0–42).
Fast Food Consumption	Continuous measure identifying the number of times respondents had fast food in the last 7 days (range: 0–50).
Body Mass Index (Obesity)	Continuous measure of respondents BMI (range: 15–98).
Health Behavior Index	Continuous measure of weighted index scores using binary versions of the health indicators above. Higher scores indicate worse health behaviors constructed in the following way:
	1. Excessive drinker if average ≥ 4 (women) or 5 (men) per instance.
	2. Smoker if respondent indicated ≥1 cigarette per day in the last 30 days
	3. Marijuana user if respondent smoked at least once in the last 30 days
	4. Physical inactivity if ≤ 7 bouts of physical activity per week
	5. Greater fast-food consumption ≥2 times per week
	6. Body Mass Index ≥30 (indicating obesity)

Source: Wave 5 of the National Longitudinal Study of Adolescent to Adult Health

#### 2.2.2 Explanatory variables

The focal explanatory variables include level of educational attainment, gender, and race. Level of education uses the maximum detail available with a total of eight categories (less than high school diploma or GED, high school diploma as reference, some postsecondary, vocational training, associate degree, bachelor's degree, master's degree, and doctorate or professional degree). Gender (male as reference versus female) and race (non-Hispanic White as reference, Black, Hispanic/Latino, and Other) are assessed using interaction models to identify differences between these key population subgroups. In addition, this study also accounts for factors known to influence returns to postsecondary educational attainment including demographic characteristics, measures of socioeconomic status, and parental background characteristics.

Demographic characteristics include age (continuous variable ranging from 33 to 44), marital status (married as reference, previously married, and never married), presence of children (no children as reference versus has children), and region of residence (South as reference, West, Midwest, and Northeast), and immigrant status (US born as reference versus non-US born).

Measures of socioeconomic status include postsecondary enrolment status (not enrolled as reference versus currently enrolled) and household income. The income variable was originally coded in categories from 1 = less than $5000 to 13 = $200,000 or more. I recoded each category to its midpoint value and divided it by 10,000 in order to use the income variable as a continuous covariate.

Parental background characteristics include family household structure (one-parent household as reference versus two-parent household), parental education (no college completion as reference versus at least one parent having completed college), family household income, and parental expectations for study respondents to complete college (not disappointed as reference, somewhat disappointed, and very disappointed). Family household income represents gross total income and is treated as a continues covariate.

#### 2.2.3 Statistical analysis

**Fig. 1:**
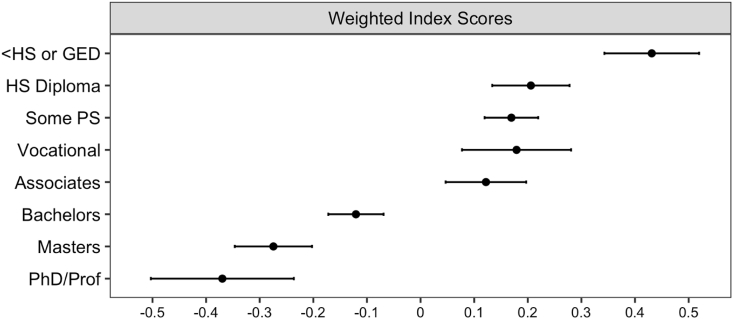
Predicted Health Behavior Index Scores. Estimates from Table 3 Model 5 are converted into predicted behavioral index scores, while holding the independent control variables constant at typical values, their average proportions or means (Fox & Andersen, 2006).

Study sample characteristics are provided for each variable included in the analyses (Table 2). Descriptive statistics are done for both the full sample as well as stratified by gender and race. As the weighted index scores are normally distributed, a series of linear regression models are estimated to assess differences in health behavior index scores (Table 3). Model 1 only includes the focal education variable to establish baseline differences in health behaviors by level of education. Model 2 adds the remaining focal explanatory variables, including gender and race. The third model includes the demographic control variables found to influence adult health behaviors. Model 4 incorporates measures of socioeconomic status. Lastly, Model 5 additionally includes parental background characteristics. The estimates from Model 5 are converted into predicted average health behavior index scores (Fig. 1), while holding the independent control variables constant at typical values, their average proportions or means (Fox & Andersen, 2006).

**Table 2:** Characteristics of the target population.

	Total Sample	Male	Female	White	Black	Hispanic
Health Behaviors						

Binge Drinking (%)	19.5	26.3	12.6	20.0	15.7	22.9
Smoking (%)	27.0	29.8	24.3	29.2	25.5	18.3
Marijuana Use (%)	20.3	24.5	16.1	20.1	24.4	18.0
Lower Physical Activity (%)	55.8	52.9	58.7	55.2	58.7	55.8
Fast Food Consumption (%)	47.8	51.5	44.2	43.9	62.9	52.5
Obesity (%)	40.5	39.2	41.9	38.1	53.0	44.1
Health Behavior Index (mean)	0.1	0.2	0.0	0.0	0.3	0.1

Level of Education (%)						

<High School or GED	9.0	11.4	6.4	8.4	11.3	11.4
High School Diploma	12.4	14.9	9.8	11.2	14.2	16.5
Some Postsecondary	25.2	26.9	23.5	23.9	30.1	28.4
Vocational Training	5.9	05.6	6.2	6.0	05.8	6.5
Associates Degree	10.3	08.7	11.9	10.8	8.9	9.2
Bachelor's Degree	23.4	22.1	24.8	25.0	17.1	19.0
Master's Degree	10.5	7.5	13.4	10.8	10.8	7.7
Doctorate/Prof Degree	3.4	2.8	3.9	3.9	1.8	1.2

Demographic Characteristics						

Female (%)	49.7			49.6	51.4	48.6
Race (%)						
Non-Hispanic White	68.5	68.7	68.3			
Black	15.2	14.7	15.7			
Hispanic/Latino	10.9	11.1	10.6			
Other	5.3	5.4	5.3			
Age (mean)	37.9	38.0	37.8	37.8	38.1	37.9
Marital Status (%)						
Married	56.4	55.6	57.2	62.0	34.2	54.2
Previously Married	16.5	15.3	17.7	16.0	18.8	18.1
Never Married	27.1	29.1	25.1	22.0	47.0	27.7
Presence of Children (%)	66.6	61.3	72.1	67.7	64.0	67.3
Region of Residence (%)						
South	41.5	41.6	41.3	37.9	64.0	43.5
West	18.3	17.7	19.0	15.5	7.8	33.6
Midwest	27.8	27.4	28.1	33.2	21.2	9.8
Northeast	12.4	13.3	11.5	13.4	7.0	13.1
Foreign Born (%)	5.9	5.8	5.9	1.2	2.3	25.1

Socioeconomic Status						

Enrolled in PS (%)	7.6	6.0	9.1	6.6	10.5	9.7
Household Income (mean)	88.0	91.1	84.8	95.0	58.0	79.9

Parental Background						

Two-Parent Household (%)	50.6	48.9	52.3	54.9	36.3	41.5
Parent Graduated College (%)	27.8	28.6	27.1	31.8	18.4	14.2
Family Household Income (mean)	7.0	7.0	7.0	7.9	4.6	5.1
Expectation to Complete College						
Not Disappointed	15.8	16.9	4.6	17.6	12.7	11.0
Somewhat Disappointed	43.2	43.0	43.3	46.7	36.2	37.1
Very Disappointed	41.0	40.0	42.0	35.6	51.1	51.9
N	11,560	5,000	6,560	6,759	2,252	1,600

Source: Wave 5 of the National Longitudinal Study of Adolescent to Adult Health.

All descriptive statistics include sampling weights to account for unequal probability of selection into the sample.

Behavior index scores represent standardized weighted values, with the mean being close to the midpoint of zero.

Both household and parental household income are measured in the tens of thousands.

**Table 3:** Linear regression of weighted health behavior index.

	Model 1	Model 2	Model 3	Model 4	Model 5
Level of Education (ref = HS Diploma)					
<High School or GED	0.30∗∗∗	0.28∗∗∗	0.25∗∗∗	0.23∗∗∗	0.23∗∗∗
	[0.19,0.41]	[0.17,0.39]	[0.14,0.36]	[0.12,0.34]	[0.12,0.34]
Some Postsecondary	-0.09	-0.08	-0.07	-0.03	-0.04
	[-0.17,0.00]	[-0.17,0.01]	[-0.16,0.02]	[-0.12,0.05]	[-0.13,0.05]
Vocational Training	-0.07	-0.05	-0.05	-0.02	-0.03
	[-0.19,0.06]	[-0.17,0.08]	[-0.17,0.08]	[-0.15,0.10]	[-0.15,0.09]
Associates Degree	-0.20∗∗∗	-0.17∗∗	-0.15∗∗	-0.08	-0.09
	[-0.31,-0.10]	[-0.28,-0.07]	[-0.26,-0.05]	[-0.19,0.02]	[-0.19,0.02]
Bachelor's Degree	-0.55∗∗∗	-0.52∗∗∗	-0.46∗∗∗	-0.33∗∗∗	-0.33∗∗∗
	[-0.63,-0.46]	[-0.61,-0.43]	[-0.55,-0.37]	[-0.42,-0.24]	[-0.42,-0.24]
Master's Degree	-0.75∗∗∗	-0.71∗∗∗	-0.65∗∗∗	-0.49∗∗∗	-0.49∗∗∗
	[-0.85,-0.65]	[-0.82,-0.61]	[-0.75,-0.55]	[-0.60,-0.39]	[-0.60,-0.38]
Doctorate/Prof Degree	-0.92∗∗∗	-0.88∗∗∗	-0.81∗∗∗	-0.59∗∗∗	-0.58∗∗∗
	[-1.07,-0.77]	[-1.03,-0.73]	[-0.96,-0.66]	[-0.74,-0.43]	[-0.74,-0.43]
Female (ref = Male)		-0.14∗∗∗	-0.15∗∗∗	-0.18∗∗∗	-0.18∗∗∗
		[-0.19,-0.09]	[-0.20,-0.10]	[-0.23,-0.13]	[-0.23,-0.13]
Race (ref = non-Hispanic White)					
Black		0.19∗∗∗	0.10∗∗	0.05	0.05
		[0.13,0.26]	[0.03,0.17]	[-0.02,0.12]	[-0.03,0.12]
Hispanic/Latino		-0.04	0.03	0.01	-0.00
		[-0.12,0.04]	[-0.06,0.11]	[-0.08,0.09]	[-0.09,0.08]
Other		-0.13∗	-0.01	-0.04	-0.05
		[-0.23,-0.03]	[-0.12,0.09]	[-0.14,0.07]	[-0.15,0.06]
Age		-0.01	-0.01	-0.01	-0.00
		[-0.02,0.00]	[-0.02,0.01]	[-0.02,0.01]	[-0.02,0.01]
R^2^	0.10	0.11	0.14	0.16	0.16

Source: Wave 5 of the 2018 National Longitudinal Study of Adolescent to Adult Health (N = 11,560).

Estimates include sampling weights to account for unequal probability of selection into the sample.

Model 1 regresses level of education on weighted index. Models 2-5 additionally include: gender, race, and age; remaining sociodemographic controls; measures of socioeconomic status; and parental background characteristics, respectively.

∗p ​< ​0.05; ∗∗p ​< ​0.01; ∗∗∗p ​< ​0.001.

**Fig. 2:**
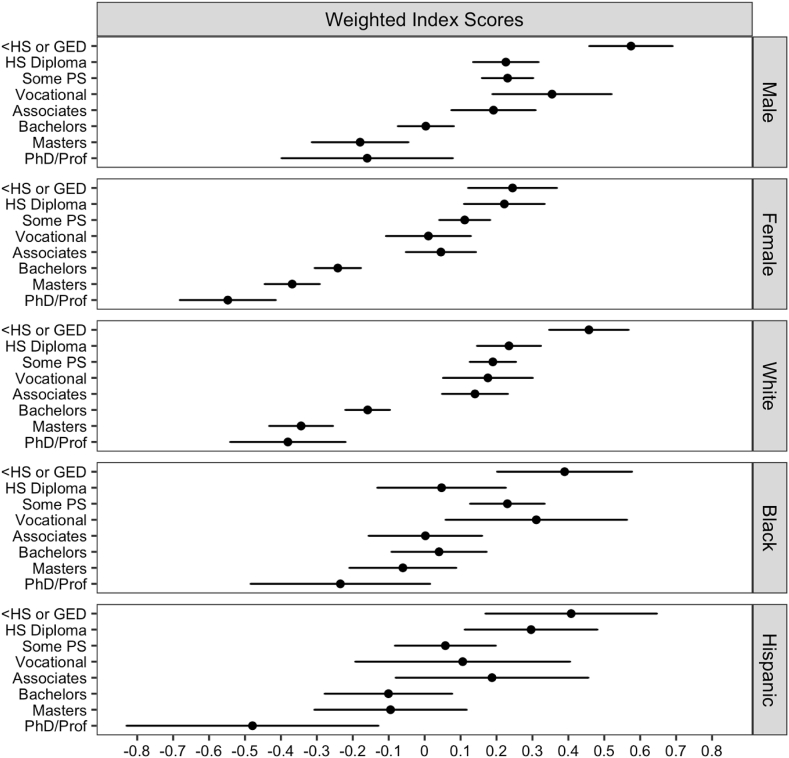
Predicted Health Behavior Index Scores by Gender and Racial Identity. Estimates from Table 4 Model 2 for each group are converted into predicted behavioral index scores, while holding the independent control variables constant at typical values, their average proportions or means (Fox & Andersen, 2006).

In order to assess differences across gender and racial identity, Table 3 includes a series of linear regression interaction models. The first model for each group includes level of education, gender, race, and age as well as the corresponding interactions. The second models include the remaining demographic characteristics, measures of socioeconomic status, and parental background characteristics. The results from the second model for each group are again used to determine predicted average index scores which are presented as a graphical display (Fig. 2). All analyses are weighted to obtain unbiased population estimates.

Missing data ranges from <0.5% for level of education, age, marital status, presence of children, and postsecondary enrolment status to approximately 2.9% for immigrant status, 6.2% for region of residence, 13.3% for parental college expectations, 17.4% for household income, and 20.2% for family household income. Missing observations are imputed using multiple imputation by chained equations (MICE) with ten replicates (Royston & White, 2011).

#### 2.2.4 Sensitivity analysis

**Appendix Fig. 1:**
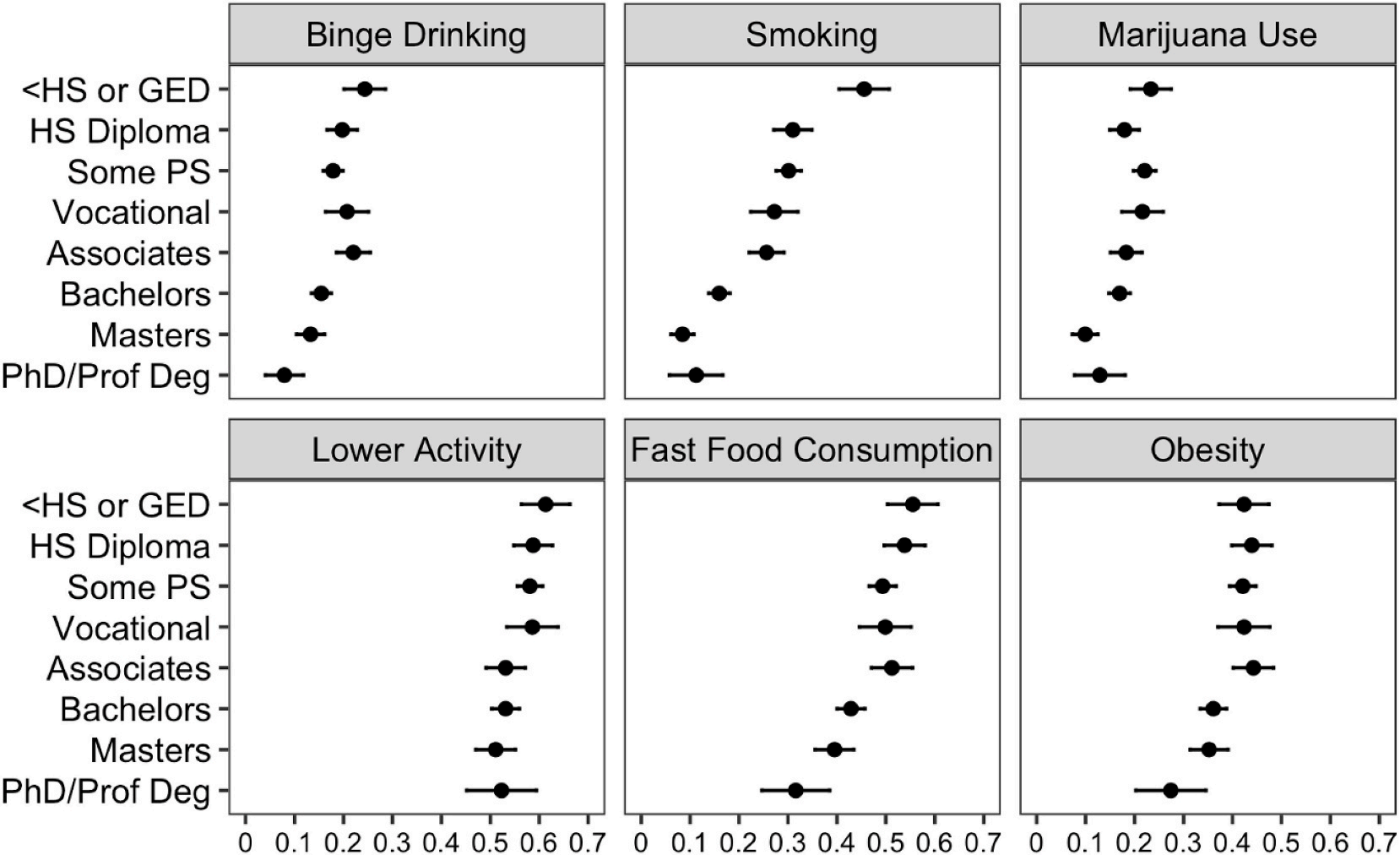
Predicted Probabilities of Individual Health Behaviors. Estimates from Appendix Table 4 are converted into predicted probabilities for each behavior, while holding the independent control variables constant at typical values, their average proportions or means (Fox & Andersen, 2006).

This study also conducts various sensitivity analyses. Appendix Table 2 includes estimates from linear regression models using an unweighted summary index of adult health behaviors (Mullan Harris, Lee, and DeLeone 2010). Appendix Table 3 assesses gender and race differences using fully adjusted stratified linear regression models. Lastly, Appendix Table 4 includes fully adjusted logistic regression estimates for each individual health behavior. To better interpret the results from these models, average adjusted probabilities are calculated from the estimated odds ratios and the findings are presented graphically (Appendix Fig. 1).

## 3 Results

Table 2 presents the prevalence of each health behavior and describes the characteristics of the target population across all variables in this study, for the full sample as well as stratified by gender and race/ethnicity population subgroups. The complex patterns observed indicate that there is substantial behavioral heterogeneity among adults 33-44 years of age. In the interest of space, I only discuss the results related to the response variables. While relatively low rates of binge drinking (20%), smoking (27%), and marijuana use are observed (20%), adults in their thirties and forties are substantially more likely to exhibit physical inactivity (56%), consume an above average amount of fast food (48%), or experience obesity (41%). The estimated gender differences in these health behaviors indicate that men are more likely to engage in unhealthy behaviors than women. Although the descriptive results show seemingly inconsistent behavior patterns across racial identity, Black Americans generally exhibit worse health behaviors than their Hispanic/Latino or non-Hispanic White counterparts.

Table 3 includes a series of linear regression models estimating differences in covariance-weighted behavior index scores. The unadjusted estimates from Model 1 indicate adults with less than a high school diploma or GED have significantly higher index scores (p<0.001), compared to high school graduates (the reference category). Adults with at least some postsecondary education reported slightly lower index scores (p<0.05), while no significant difference is observed among adults with a vocational certificate. The remaining levels of education have increasingly lower health behavior index scores (p<0.001). These estimates remain largely the same after controlling for gender and race. While the education coefficients are slightly attenuated in the fully adjusted regression model, these estimates also indicate that the variables assessed in this study explain approximately 16% of the difference in health behavior index scores.

Fig. 1 includes fully adjusted predicted health behavior index scores. While adults with less than a high school diploma or GED continue to have the highest predicted index scores (which indicates increased engagement in unhealthy behaviors), those with either a high school diploma, some postsecondary, vocational certificate, or associates degree are not found to significantly differ. The results indicate a decrease in unhealthy behaviors among those with a bachelor's degree and again among those with either a graduate or professional degree, when compared to adults with any other level of education.

Table 4 includes a series of interaction models for both gender and race/ethnicity population subgroups. Compared to those with a high school diploma, men with less than a high school diploma or GED exhibit significantly higher health behavior index scores, while their female counterparts do not. Moreover, the benefit of higher education is only observed among men with at least a bachelor's degree, where women begin to exhibit better health behaviors at the vocational certificate level. These differences, as well as the steeper overall slope across levels of education, suggest women benefit more from higher levels of postsecondary education and supports a resource substitution hypothesis.

**Table 4:** Linear regression of weighted health behavior index, gender and race interactions.

	Gender		Race	
	Model 1	Model 2	Model 1	Model 2
Level of Education (ref = HS Diploma)				
<High School or GED	0.39∗∗∗	0.35∗∗∗	0.30∗∗∗	0.22∗∗
	[0.25,0.54]	[0.20,0.49]	[0.16,0.44]	[0.08,0.36]
Some Postsecondary	-0.05	0.01	-0.08	-0.05
	[-0.17,0.06]	[-0.11,0.12]	[-0.19,0.03]	[-0.15,0.06]
Vocational Training	0.10	0.13	-0.07	-0.06
	[-0.09,0.29]	[-0.06,0.32]	[-0.23,0.08]	[-0.21,0.09]
Associates Degree	-0.15	-0.03	-0.17∗∗	-0.10
	[-0.30,0.00]	[-0.18,0.11]	[-0.30,-0.05]	[-0.22,0.03]
Bachelor's Degree	-0.41∗∗∗	-0.22∗∗∗	-0.58∗∗∗	-0.39∗∗∗
	[-0.53,-0.30]	[-0.34,-0.10]	[-0.69,-0.47]	[-0.50,-0.28]
Master's Degree	-0.65∗∗∗	-0.41∗∗∗	-0.81∗∗∗	-0.58∗∗∗
	[-0.82,-0.49]	[-0.57,-0.24]	[-0.93,-0.68]	[-0.71,-0.45]
Doctorate/Prof Degree	-0.68∗∗∗	-0.39∗∗	-0.92∗∗∗	-0.62∗∗∗
	[-0.94,-0.42]	[-0.64,-0.13]	[-1.10,-0.74]	[-0.80,-0.43]
Female (ref = Male)	0.02	-0.00	-0.14∗∗∗	-0.19∗∗∗

Race (ref = non-Hispanic White)	[-0.12,0.16]	[-0.15,0.14]	[-0.19,-0.092]	[-0.24,-0.14]
Black	0.19∗∗∗	0.04	-0.03	-0.19
	[0.12,0.26]	[-0.03,0.11]	[-0.22,0.16]	[-0.39,0.01]
Hispanic/Latino	-0.04	-0.00	0.01	0.06
	[-0.12,0.04]	[-0.09,0.09]	[-0.18,0.20]	[-0.14,0.27]
Age	-0.01	-0.00	-0.01	-0.00
	[-0.02,0.00]	[-0.02,0.01]	[-0.02,0.00]	[-0.02,0.01]
<HS or GED∗Female	-0.29∗∗	-0.33∗∗		
	[-0.51,-0.07]	[-0.54,-0.11]		
Some Postsecondary∗Female	-0.08	-0.12		
	[-0.26,0.09]	[-0.29,0.06]		
Vocational∗Female	-0.32∗	-0.34∗∗		
	[-0.57,-0.07]	[-0.59,-0.09]		
Associates∗Female	-0.10	-0.14		
	[-0.31,0.11]	[-0.35,0.07]		
Bachelors∗Female	-0.24∗∗	-0.24∗∗		
	[-0.41,-0.07]	[-0.41,-0.07]		
Masters∗Female	-0.16	-0.18		
	[-0.37,0.05]	[-0.39,0.02]		
Doctorate/Prof Degree∗Female	-0.40∗	-0.38∗		
	[-0.71,-0.09]	[-0.68,-0.08]		
<HS or GED∗Black			0.08	0.12
			[-0.21,0.37]	[-0.17,0.41]
<HS or GED∗Hispanic			-0.17	-0.11
			[-0.49,0.15]	[-0.44,0.21]
Some Postsecondary∗Black			0.21	0.23
			[-0.02,0.43]	[-0.00,0.46]
Some Postsecondary∗Hispanic			-0.20	-0.19
			[-0.45,0.05]	[-0.45,0.06]
Vocational∗Black			0.35∗	0.32
			[0.01,0.69]	[-0.02,0.66]
Vocational∗Hispanic			-0.15	-0.13
			[-0.54,0.23]	[-0.51,0.25]
Associates∗Black			0.01	0.05
			[-0.26,0.27]	[-0.22,0.32]
Associates∗Hispanic			-0.02	-0.01
			[-0.36,0.33]	[-0.36,0.33]
Bachelors∗Black			0.37∗∗	0.39∗∗
			[0.14,0.61]	[0.14,0.63]
Bachelors∗Hispanic			0.06	-0.00
			[-0.21,0.33]	[-0.28,0.27]
Masters∗Black			0.48∗∗∗	0.47∗∗∗
			[0.22,0.74]	[0.21,0.73]
Masters∗Hispanic			0.20	0.19
			[-0.10,0.50]	[-0.11,0.49]
Doctorate/Prof Degree∗Black			0.40∗	0.33
			[0.06,0.75]	[-0.02,0.7]
Doctorate/Prof Degree∗Hispanic			-0.08	-0.16
			[-0.51,0.34]	[-0.59,0.27]
R^2^	0.12	0.16	0.12	0.16

Source: Wave 5 of the 2018 National Longitudinal Study of Adolescent to Adult Health (N = 11,560).

Estimates include sampling weights to account for unequal probability of selection into the sample.

Model 1 includes level of education, gender, race, and age, as well as the corresponding interaction.

Model 2 additionally include the remaining control variables assessed in this study.

∗p ​< ​0.05; ∗∗p ​< ​0.01; ∗∗∗p ​< ​0.001.

Interesting differences are also observed by race. The pattern observed among White adults is comparable to that of the Hispanic population, whereby lower index scores are only observed among those with at least a bachelor's degree, compared to those with a high school diploma. However, Black Americans only exhibit lower health behavior index scores at the doctoral or professional degree level. For an alternative means of interpretation, the fully adjusted interaction models are converted into predicted health behavior index scores, while holding the independent control variables constant at typical values, their average proportions or means (Fox & Andersen, 2006). The predicted index scores, along with corresponding 95% confidence intervals, are plotted in Fig. 2.

## 4 Discussion

Despite the strong link between a bachelor's degree and adult health behaviors, we know little about the behavioral patterns among those with lower-level postsecondary credentials. These credentials represent profoundly different levels of human capital accumulation, which lead to comparably varied life trajectories. As a result, this study examines differences in adult health behaviors across detailed levels of postsecondary education using covariance-weighted behavioral index scores. The analyses also substantially extend prior work by providing health behavior estimates both in aggregate and across gender and race/ethnicity population subgroups. The main findings indicate that higher levels of education led to healthier behaviors in adulthood; however, generally only at the bachelor's degree level and beyond, with no improvements in adult health behaviours among those with sub-BA levels of education. These estimates also fill an important gap in the literature as existing education-health studies generally group postsecondary credential into a limited number of categories (Case & Deaton, 2021; Lawrence, 2017; Zheng, 2017).

The observed behavioral index scores indicate substantial heterogeneity in adult health behaviors. While adults with either less than a high school diploma or GED have the worst health behaviors, those with either a high school diploma, some postsecondary, a vocational certificate, or an associate degree do not significantly differ. However, significantly better health behaviors are observed among those with a bachelor's degree and again among those with either a graduate or professional degree, when compared to adults with any other level of education. The lack of behavioral improvements among the sub-BA levels of education suggests these lower-level credentials do not provide enough human capital, economic resources, and psychosocial abilities necessary to enable healthier lifestyles in adulthood. This has important implications as the vast majority of young adults are pursuing at least some form of postsecondary education, but improved health behaviors seem to be concentrated at the highest rungs of that education latter perpetuating social disadvantages.

The findings also show that women experience steeper education-health gradients, compared to men. For example, women that have completed any type of postsecondary education have significantly lower predicted behavioral index scores, compared to those with a high school diploma. In comparison, men only experience returns to a postsecondary education at the bachelor's degree level and beyond. These estimates support a process of resource substitution, as women generally have fewer alternative health-promoting socioeconomic resources (Ross & Mirowsky, 2010; Vable et al., 2018).

The predicted index scores also reveal interesting behavioral patterns across racial identity as White and Hispanic/Latino Americans have comparable health lifestyles across levels of postsecondary education; however, Black Americans were found to experience no returns except at the doctoral or professional degree level. This is consistent with other recent studies which have found flatter education-health gradients among Black adults (Bell et al., 2018; Kroeger & Frank, 2018; Vable et al., 2018). These estimates may reflect the higher levels of stress and economic disadvantages experienced by Black Americans (Goldman et al., 2006; Simons et al., 2020; Turra & Goldman, 2007). It is also indicative of a resource multiplication process whereby socially advantaged groups gain more from education as their social and economic resources multiply to perpetuate and enhance their advantage (Ross & Mirowsky, 2006).

### 4.1 Limitations

As one of the first studies to identify disparities in health behaviors across the entire postsecondary education gradient, this work has limitations that future research could address. While I control for differences in demographic characteristics, there may be other confounders that were missed. Future research could further examine social and contextual determinants of health behaviors in adulthood. As potential selection effects were not specifically addressed, future studies may want to account for health differences in adolescence when estimating the association between education and adult health behaviors. Further research on these important relationships will continue to help us understand inequalities in adult health behaviors.

### 4.2 Conclusion

In this article, I describe large behavioral disparities among lower level postsecondary credentials which persist even after controlling for various demographic characteristics, measures of socioeconomic status, and parental background characteristics. The findings highlight the complex and profound association between a postsecondary education and adult health behaviors. As such, I strongly encourage scholars and policymakers to continue to examine how differentiation in postsecondary education may impact various health outcomes. These findings are also informative for policy initiatives and any potential targeted public health interventions.

## Financial disclosures

This study was not supported by any funding sources.

## Author statement

Anthony Jehn: Conceptualization, Formal Analysis, Software, Visualizations, Writing – Original and Revised Drafts.

## Declaration of competing interest

The author declares no conflict of interest.

## Figures and Tables

**Appendix Table 1:** Tetrachoric Correlations Between Binary Health Behaviors

	Drinking	Smoking	Marijuana	Inactivity	Fast Food	Obesity
Drinking	1.00					
Smoking	0.37	1.00				
Marijuana	0.29	0.46	1.00			
Inactivity	0.01	0.07	-0.05	1.00		
Fast Food	0.08	0.07	0.02	0.16	1.00	
Obesity	0.06	-0.02	-0.09	0.19	0.27	1.00

Source: Wave 5 of the 2018 National Longitudinal Study of Adolescent to Adult Health (N = 11,560).

**Appendix Table 2:** Linear Regression of Summary Health Behavior Index

	Model 1	Model 2	Model 3	Model 4	Model 5
Level of Education (ref = HS Diploma)					
<High School or GED	0.39∗∗∗	0.37∗∗∗	0.33∗∗∗	0.29∗∗∗	0.29∗∗∗
Some Postsecondary	-0.13∗	-0.12∗	-0.11	-0.06	-0.06
Vocational Training	-0.11	-0.09	-0.08	-0.05	-0.06
Associates Degree	-0.27∗∗∗	-0.24∗∗∗	-0.22∗∗	-0.12	-0.12
Bachelor's Degree	-0.74∗∗∗	-0.70∗∗∗	-0.63∗∗∗	-0.46∗∗∗	-0.45∗∗∗
Master's Degree	-0.99∗∗∗	-0.95∗∗∗	-0.87∗∗∗	-0.65∗∗∗	-0.64∗∗∗
Doctorate/Prof Degree	-1.22∗∗∗	-1.17∗∗∗	-1.08∗∗∗	-0.78∗∗∗	-0.77∗∗∗
Female (ref = Male)		-0.16∗∗∗	-0.17∗∗∗	-0.22∗∗∗	-0.22∗∗∗
Race (ref = Non-Hispanic White)					
Black		0.25∗∗∗	0.13∗∗	0.07	0.06
Hispanic/Latino		-0.06	0.02	0.01	-0.01
Other		-0.16∗	0.00	-0.03	-0.04
Age			-0.01	-0.00	-0.00
Marital Status (ref = Married)					
Previously Married			0.32∗∗∗	0.22∗∗∗	0.22∗∗∗
Never Married			0.35∗∗∗	0.25∗∗∗	0.25∗∗∗
Presence of Children (ref = No Children)			0.07	0.07	0.07
Region of Residence (ref = South)					
West			-0.29∗∗∗	-0.24∗∗∗	-0.22∗∗∗
Midwest			0.01	0.01	0.01
Northeast			-0.33∗∗∗	-0.28∗∗∗	-0.28∗∗∗
Non-US Born (ref = US Born)			-0.23∗∗∗	-0.20∗∗	-0.21∗∗
Enrolled in PS (ref = Not Enrolled)				-0.21∗∗∗	-0.21∗∗∗
Household Income				-0.03∗∗∗	-0.03∗∗∗
Two-Parent Household					0.03
Parent Graduated College					-0.10∗∗
Family Household Income					-0.00
Expectation to Complete College (ref = Not Disappointed)					
Somewhat Disappointed					0.08
Very Disappointed					0.09
R^2^	0.11	0.12	0.15	0.16	0.17

Source: Wave 5 of the 2018 National Longitudinal Study of Adolescent to Adult Health (N = 11,560).

Estimates include sampling weights to account for unequal probability of selection into the sample.

∗p ​< ​0.05; ∗∗p ​< ​0.01; ∗∗∗p ​< ​0.001.

**Appendix Table 3:** Linear Regression of Weighted Health Behavior Index, Stratified by Gender and Race

	Male	Female	White	Black	Hispanic
Level of Education (ref = HS Diploma)					
<High School or GED	0.35∗∗∗	0.02	0.22∗∗	0.36∗∗	0.10
Some Postsecondary	-0.01	-0.11	-0.04	0.16	-0.26∗
Vocational Training	0.12	-0.20∗	-0.06	0.27	-0.23
Associates Degree	-0.04	-0.17∗	-0.08	-0.10	-0.12
Bachelor's Degree	-0.27∗∗∗	-0.43∗∗∗	-0.37∗∗∗	-0.08	-0.47∗∗∗
Master's Degree	-0.45∗∗∗	-0.55∗∗∗	-0.56∗∗∗	-0.18	-0.46∗∗
Doctorate/Prof Degree	-0.46∗∗∗	-0.71∗∗∗	-0.59∗∗∗	-0.33∗	-0.92∗∗∗
Female (ref = Male)			-0.18∗∗∗	-0.14∗	-0.25∗∗
Race (ref = Non-Hispanic White)					
Black	0.03	0.05			
Hispanic/Latino	0.03	-0.04			
Other	-0.06	-0.05			
Age	-0.02	0.01	0.00	-0.02	-0.03
Marital Status (ref = Married)					
Previously Married	0.21∗∗∗	0.15∗∗∗	0.22∗∗∗	-0.02	0.17
Never Married	0.18∗∗∗	0.22∗∗∗	0.23∗∗∗	0.00	0.27∗∗
Presence of Children (ref = No Children)	0.06	0.01	-0.00	0.05	0.23∗∗
Region of Residence (ref = South)					
West	-0.11∗	-0.16∗∗∗	-0.18∗∗∗	-0.19	-0.03
Midwest	0.01	0.04	-0.01	0.09	0.28
Northeast	-0.19∗∗∗	-0.18∗∗∗	-0.20∗∗∗	-0.12	-0.06
Non-US Born (ref = US Born)	-0.17	-0.17∗∗	-0.10	-0.41∗∗	-0.03
Enrolled in PS (ref = Not Enrolled)	-0.25∗∗	-0.12∗	-0.21∗∗∗	-0.04	-0.14
Household Income	-0.02∗∗∗	-0.03∗∗∗	-0.03∗∗∗	-0.021∗∗∗	-0.02∗
Two-Parent Household	-0.01	0.04	0.05	-0.01	-0.09
Parent Graduated College	-0.01	-0.12∗∗∗	-0.07∗	-0.06	0.01
Family Household Income	-0.00	0.00	0.00	-0.00	0.01
Expectation to Complete College (ref = Not Disappointed)					
Somewhat Disappointed	0.09	0.04	0.07	0.12	0.09
Very Disappointed	0.11∗	0.04	0.09	0.15	0.03
R^2^	0.13	0.18	0.17	0.09	0.12
N	5000	6560	6759	2252	1600

Source: Wave 5 of the 2018 National Longitudinal Study of Adolescent to Adult Health.

Estimates include sampling weights to account for unequal probability of selection into the sample.

∗p ​< ​0.05; ∗∗p ​< ​0.01; ∗∗∗p ​< ​0.001.

**Appendix Table 4:** Logistic Regression of Individual Health Behaviors

	Binge Drinking	Smoking	Marijuana Use	Lower Physical Activity	Fast Food Consumption	Body Mass Index (Obesity)
Level of Education (ref = HS Diploma)	1.31	1.86∗∗∗	1.39∗	1.11	1.07	0.94
<High School or GED	0.88	0.96	1.29∗	0.97	0.84	
Some Postsecondary	1.06	0.83	1.26	0.99	0.85	0.93
Vocational Training	1.15	0.77∗	1.02	0.80	0.90	0.94
Associates Degree	0.74∗	0.42∗∗∗	0.93	0.80∗	0.64∗∗∗	1.01
Bachelor's Degree	0.62∗∗	0.21∗∗∗	0.50∗∗∗	0.73∗	0.56∗∗∗	0.72∗∗
Master's Degree	0.35∗∗∗	0.28∗∗∗	0.68	0.77	0.40∗∗∗	0.69∗∗
Doctorate/Prof Degree	0.88	0.96	1.29∗	0.97	0.84	0.48∗∗∗
Female (ref = Male)	0.42∗∗∗	0.82∗∗	0.60∗∗∗	1.30∗∗∗	0.73∗∗∗	0.93
Race (ref = Non-Hispanic White)						
Black	0.67∗∗∗	0.50∗∗∗	1.15	0.98	1.69∗∗∗	1.46∗∗∗
Hispanic/Latino	1.31∗	0.47∗∗∗	0.85	0.93	1.29∗∗	1.28∗
Other	1.03	0.82	0.69∗	1.02	1.28∗	0.85
Age	0.94∗∗	1.00	0.96∗	1.02	1.00	1.03∗
Marital Status (ref = Married)						
Previously Married	1.63∗∗∗	1.97∗∗∗	1.70∗∗∗	0.86	0.98	0.91
Never Married	1.67∗∗∗	1.92∗∗∗	1.76∗∗∗	0.92	1.00	0.92
Presence of Children (ref = No Children)	1.09	1.17∗	0.89	1.03	1.18∗	1.01
Region of Residence (ref = South)						
West	0.73∗∗	0.75∗∗	1.96∗∗∗	0.78∗∗	0.60∗∗∗	0.77∗∗
Midwest	1.21∗	1.15	1.33∗∗	0.92	0.82∗∗	0.92
Northeast	0.89	0.85	1.27∗	0.89	0.38∗∗∗	0.89
Non-US Born (ref = US Born)	0.79	0.67∗	0.53∗∗	1.17	1.04	0.64∗∗
Enrolled in PS (ref = Not Enrolled)	0.68∗∗	0.70∗∗	0.79	0.80∗	0.91	1.00
Household Income	1.01	0.95∗∗∗	0.96∗∗∗	0.97∗∗∗	0.98∗∗∗	0.96∗∗∗
Two-Parent Household	1.03	0.91	0.84∗	1.05	1.19∗∗	1.10
Parent Graduated College	0.85	0.91	1.20∗	0.94	0.87∗	0.86∗
Family Household Income	1.01	1.00	1.02∗∗∗	1.00	0.98∗∗∗	0.99
Expectation to Complete College (ref = Not Disappointed)						
Somewhat Disappointed	0.98	1.14	1.26∗	1.14	1.05	0.93
Very Disappointed	1.03	1.03	1.16	1.13	1.11	1.00

Source: Wave 5 of the 2018 National Longitudinal Study of Adolescent to Adult Health (N = 11,560).

Estimates include sampling weights to account for unequal probability of selection into the sample.

∗p ​< ​0.05; ∗∗p ​< ​0.01; ∗∗∗p ​< ​0.001.
